# Change in Dielectric Properties in the Microwave Frequency Region of Polypyrrole–Coated Textiles during Aging

**DOI:** 10.3390/ma9070609

**Published:** 2016-07-22

**Authors:** Eva Hakansson, Akif Kaynak, Abbas Kouzani

**Affiliations:** School of Engineering, Deakin University, Geelong, VIC 3216, Australia; evas.mejl@gmail.com (E.H.); abbas.kouzani@deakin.edu.au (A.K.)

**Keywords:** microwave, dielectric constant, aging

## Abstract

Complex permittivity of conducting polypyrrole (PPy)-coated Nylon-Lycra textiles is measured using a free space transmission measurement technique over the frequency range of 1–18 GHz. The aging of microwave dielectric properties and reflection, transmission and absorption for a period of 18 months is demonstrated. PPy-coated fabrics are shown to be lossy over the full frequency range. The levels of absorption are shown to be higher than reflection in the tested samples. This is attributed to the relatively high resistivity of the PPy-coated fabrics. Both the dopant concentration and polymerisation time affect the total shielding effectiveness and microwave aging behaviour. Distinguishing either of these two factors as being exclusively the dominant mechanism of shielding effectiveness is shown to be difficult. It is observed that the PPy-coated Nylon-Lycra samples with a *p*-toluene sulfonic acid (*p*TSA) concentration of 0.015 M and polymerisation times of 60 min and 180 min have 37% and 26% decrease in total transmission loss, respectively, upon aging for 72 weeks at room temperature (20 °C, 65% Relative humidity (RH)). The concentration of the dopant also influences the microwave aging behaviour of the PPy-coated fabrics. The samples with a higher dopant concentration of 0.027 mol/L *p*TSA are shown to have a transmission loss of 32.6% and 16.5% for short and long polymerisation times, respectively, when aged for 72 weeks. The microwave properties exhibit better stability with high dopant concentration and/or longer polymerization times. High *p*TSA dopant concentrations and/or longer polymerisation times result in high microwave insertion loss and are more effective in reducing the transmission and also increasing the longevity of the electrical properties.

## 1. Introduction

Conducting polymers such as polypyrrole and polyaniline, when used in conjunction with suitable substrates, exhibit high radiation absorption and shielding in the microwave frequency region [1,2]. Wide-ranging modulation of electrical and electromagnetic properties of conducting polymer films can be utilised effectively when they are coated on flexible and strong substrates such as textiles. The intricate structures and fibrous surfaces of textiles enable deep penetration of the polymer into the interstices of the flexible fibre assemblies. Conducting polymer-coated textiles have potential applications as frequency-selective absorbers of electromagnetic radiation, and as electromagnetic shields for modifying and controlling the physical indoor wireless channel for the reduction of multipath and electromagnetic interference. Conducting polymers possess wide-ranging electrical resistivity and electromagnetic reflective and absorptive properties that can be controlled during the polymerisation stage, by the type and concentrations of the monomer, dopant, oxidant, solvent, polymerisation time, temperature and type of substrate. Moreover, the sensitivity of the electrical properties to the external effects such as temperature and radiation indicates potential applications in the fields of electromagnetic interference and microwave absorption. Although conducting polymer composites have various potential applications in the microwave frequency region, the degradation of electrical properties has been one of the main drawbacks of conducting polymers and conducting polymer coatings [3]. The degradation or chemical aging causes an increase in the electrical resistivity which in turn changes the nature of the interaction of the material with electromagnetic waves and manifests as changes in the complex permittivity, microwave reflection, transmission and absorption behaviour in conductive polymer films and conductive textiles. As the material loses conductivity with aging, microwave transmission increases while reflection decreases throughout the microwave frequency range. The aging of these properties should be characterised and taken into account before attempting any applications of these materials in the microwave frequency region. In a study of the electrical degradation of free-standing conducting polymers [4,5], it was observed that highly *p*-toluene sulfonic acid (*p*TSA)-doped polypyrrole (PPy) films had good electrical stability, holding their high microwave reflectivity during the aging period, whereas lightly doped films were not as stable, exhibiting considerable loss of reflectivity. The increased concentration of the dopant *p*TSA had a positive influence on the degradation. The degradation in conducting polymers upon aging was mainly attributed to oxygen attack and the formation of carbonyl species in the polymer backbone [5], but heat, light, mechanical stress, ozone, moisture and atmospheric pollutants also contributed [6]. The formation of carbonyl species shortens the conjugation length in the material and hence decreases conductivity. The stabilising effect of the dopant *p*TSA against thermal degradation was also observed in another aging study on PPy-*p*TSA–coated polyethyleneterephthalate (PET) fabrics. The fabric gained less than an order of magnitude in resistivity upon aging for 18 months at room temperature [7]. Various studies of electrical aging of *p*TSA-doped PPy films have been carried out at room temperature and elevated temperatures in an effort to understand the kinetics of the degradation and to foresee the long-term performance of the free-standing PPy films and coatings on textiles. Aging studies at elevated temperatures showed that the kinetics of degradation deviated from the first-order kinetics that had been predicted by Chen at al [8] earlier. Longer-term aging at elevated temperatures showed that the degradation of conductivity exhibited a non-linear relationship with the natural logarithm of normalised electrical conductivity, with the aging period implying a multi-order aging process [4].

There have been various studies on the thermal degradation and kinetics of degradation of conducting polymers [4,5,8,9,10]; however, there have not been any reports on the aging behaviour of dielectric properties, reflection, transmission and absorption in the microwave regime. In this paper, we present the complex permittivity of conducting polypyrrole (PPy)-coated Nylon-Lycra textiles by using a free space transmission measurement technique over the frequency range 1–18 GHz and also report the changes in permittivity and transmission losses over a period of 72 weeks at room temperature.

## 2. Experiments

Chemical polymerisation of pyrrole in aqueous solution was used as the coating method. Oxidation of the monomer leads to direct deposition of the conducting polymer on the surface of the textile as well precipitation of insoluble polymers in solution (bulk polymerisation) [11]. The direct deposition on the surface produces a tightly bound coating whereas subsequent deposition of the bulk polymerised nodular particles are loosely bound and should be washed off after the completion of the coating. The major advantages of the chemical over the electrochemical procedure are low cost, ease of coating, and possibility to make large sample sizes. Oxidation of pyrrole monomer was achieved using ferric chloride hexahydrate (FeCl_3_). The pyrrole monomer, dopant *p*-toluene sulfonic acid (*p*TSA) and the oxidising agent ferric chloride were obtained from Aldrich (Sydney, Australia). A double-sided basket-weave Nylon-Lycra^®^ (Spotlight, Geelong, Australia) with an average Lycra content of 20% was used as substrate. The tests have been carried out on the ‘non-textured’ Nylon-Lycra, which was chosen due to even coatings being achieved and resulting high conductivities. Although we coated various textiles with PPy, including wool, cotton, PET and Nylon-Lycra, the latter responded well to the polymerisation, resulting in more even coatings and lower resistivity conductive textiles. The fabric samples were cut to size, dried in a lab oven (Binder FED 115, Tuttlingen, Germany) at 105 °C and allowed to cool to room temperature. The monomer, wetting agent, fabric samples, dopant and solvent were mixed thoroughly. The textile substrate was thoroughly soaked for approximately 10 min in the solution. Then the oxidising agent was added to the solution to initiate polymerisation followed by vigorous stirring for 30 s. The reaction vessel was stirred for 10 s per 30 min. The polymerisation time varied between 5 and 300 min with the reaction volume fixed at 1500 mL for the large samples (500 mm square) and 600 mL for the small samples (305 mm square), respectively. After a certain reaction time, the sample was removed and washed with copious amounts of water. Washing was continued until most of the bulk polymerised polymer nodules were removed from the fabric. Samples were then dried in a laboratory oven at 25 °C for 8 h. Finally, conductive textiles were cut to size and stored flat in an airing cabinet in a conditioned laboratory at 20 °C, 65% RH prior to and between all measurements.

**Figure 1:**
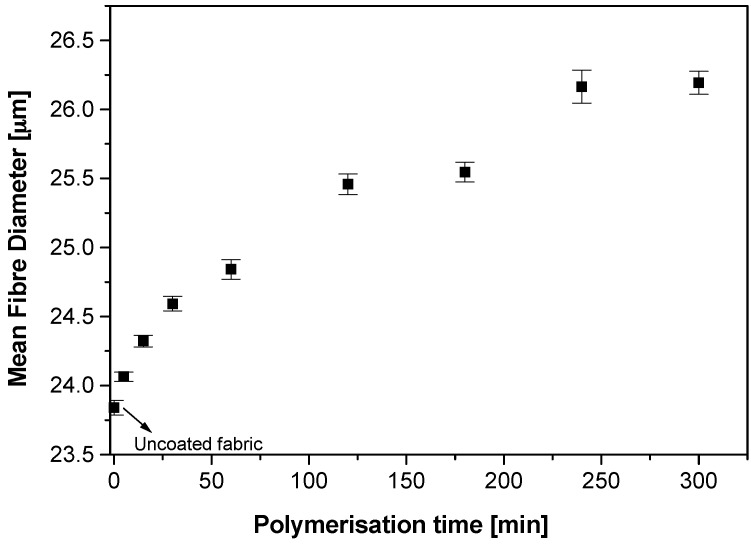
The variation of the diameter of Nylon-Lycra fibre with polymerisation time. Dopant concentration 0.018 mol/L *p*TSA.

Polypyrrole deposition on fabrics with respect to polymerisation time and the dopant concentration were examined by using optical fibre diameter analysis (OFDA). As the reaction time and/or reactant concentrations increase, the uncoated white fabric changes from lighter tones to darker tones and finally turns deep black in colour after prolonged reaction times. A gradation of tones of fabric from light tones to deep black was observed with polymerisation times 5, 15, 30, 60, 120 and 300 min. The deepening of the tone was accompanied by fibre diameter increase due to the increase in the thickness of the coating with the polymerisation time. Fibre diameter measurements of the uncoated and coated fibres were done by using an using an OFDA 2000 (BSC Electronics, Pty Ltd., Ardross, Australia) that utilises optical image analysis to gauge the fibre diameters of 2 mm snippets of fibres between two glass slides. In this process a minimum of 35,000 fibres from each sample were measured and averaged. The fibre diameter analysis showed that the mean diameter of the pristine nylon-Lycra fibres was 23.84 ± 0.1 µm. The mean fibre diameter increased with reaction time as the coating deposited on the fabric. The rate of mean fibre diameter increase was higher in the initial stages of polymerisation and the rate gradually decreased with the increase in polymerisation time. The deposition was higher at the initial stages but as the reactants depleted with time the deposition rate decreased (Figure 1).

**Figure 2:**
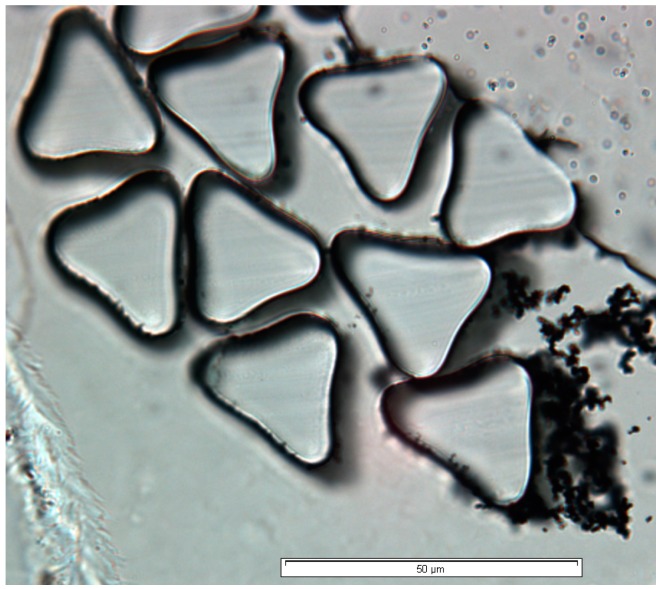
Optical transmission microscopy image of microtome sections of sample fibres of PPy-*p*TSA coated Nylon-Lycra fabrics, coated for 120 min of polymerisation time.

These observations indicate that the PPy coating thickness ranged from 0.15 to 2.4 µm with the polymerisation time ranging from 5 to 300 min (Figure 1). On the other hand, the variation of dopant concentration at a fixed polymerisation time had no significant effect on the mean fibre diameter. In addition to OFDA measurements, optical transmission microscopy analysis was performed on 8 µm microtome sections of polypyrrole coated fabric mounted in Technovit 7100 resin (Emgrid, Gulfview Heights, Australia) using a camera attached to an Olympus BX51 microscope (Melbourne, Australia). The analysis showed that the polypyrrole coating covered the entire circumference of each fibre (Figure 2). The coating appeared to be adherent to the fibre.

The increase in coating thickness with polymerisation time was accompanied with an increase in the rate of deposition of bulk polymerised nodular polymer particles on the fibres and the interstices of the fabric structure. As mentioned earlier, these polymer particles are loosely bound, undesirable and removed from the surface by a thorough washing. Some of these bulk polymerised depositions can be seen on the lower right section of Figure 2.

**Figure 3:**
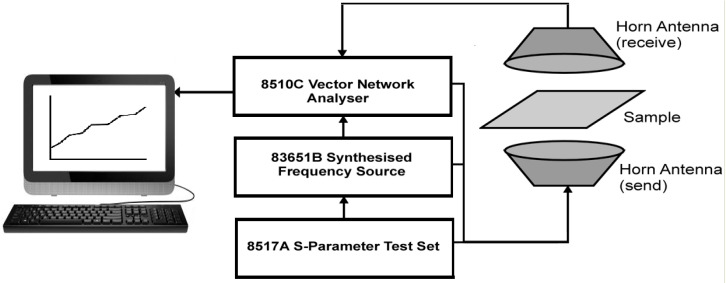
Schematic of free space transmission set-up.

Microwave measurements were carried out by free-space transmission measurement, which is a non-contact free space method that is ideally suited to determine dielectric characteristics for thin, flexible samples such as the conducting polymer coated fabrics. Figure 3 shows a schematic of the free space transmission set-up. The sample is placed flat in the line of radiation between two antennae. A radiation output system then generates a swept signal across a pre-set frequency range and a network analyzer collects the data from the measurement. The transmission and/or reflection of the sample can be determined from the scattering parameters S_11_ and S_21_. The S_11_ and S_21_ can be converted into magnitude and phase of the reflection, transmission, and the permittivity of the device under test. The relative permittivity of a material consists of a real part ($\varepsilon^{\prime }$), related to the amount of polarisation, and an imaginary part ($\varepsilon^{\prime \prime }$), related to dissipation of energy in the material

(1) $$\varepsilon_{r}={\varepsilon^{\prime }}_{r}+i\varepsilon_{r}^{\prime \prime }$$

As an electromagnetic wave travelling in free space encounters a material, depending on the electrical properties and the thickness of the material, some reflection, transmission and absorption occur. Considering a material/air interface, the reflection coefficient Γ is defined as the fraction of radiation that is reflected from the impinging surface and can be expressed in terms of relative permittivity and permeability as

(2) $$\Gamma =\frac{Z_{2}-Z_{1}}{Z_{2}+Z_{1}}=\frac{\sqrt{\frac{\mu_{r}}{\varepsilon_{r}}}-1}{\sqrt{\frac{\mu_{r}}{\varepsilon_{r}}}+1}$$

where *Z*_1_ is characteristic impedance of free space and *Z*_2_ is the characteristic impedance of the medium encountered. Whereas the transmission coefficient, *T*, is the ratio of transmitted electric field strength to the incident electric field strength and is given by:

(3) $$T=e^{-d\sqrt{-\omega^{2}\varepsilon_{r}\mu_{r}}}=e^{-i\left(\frac{\omega }{c}\right)d\sqrt{\varepsilon_{r}\mu_{r}}}$$

where *ω = 2πf* is the angular frequency in Hz at a specific frequency *f*, *μ*_r_ is the relative permeability (since the conductive fabrics under investigation was non-magnetic, relative permeability was set to 1). The permeability of the free space is *μ*_0_ = 4π × 10^−7^ H/m, *d* is the sample thickness and *c* is the speed of light.

The reflection and transmission coefficients and *R*(dB) and *T*(dB) are related as follows:

(4) $$R(\mathrm{dB})=20\log\left(\Gamma_{in}\right)$$

and

(5) T(dB)=20logT

The percentages of reflection *R*(%) and transmission *T*(%) were calculated using the relationships

(6) $$R(\%)=100\times 10^{\left(R(\mathrm{dB})/10\right)}$$

and

(7) $$T(\%)=100\times 10^{\left(T(\mathrm{dB})/10\right)}$$

The absorption percentage *A*(%) was calculated by using

(8) A(%)=100−T(%)−R(%)

The shielding effectiveness of the conducting fabrics was obtained by determining the percentage of the incident radiation that is not transmitted through the fabric, in other words, radiation that is both absorbed and reflected by the material. This is often referred to as total transmission loss.

An Agilent Technology 8510C vector network analyzer (Melbourne, Australia) was used to perform the measurements at microwave frequencies. The 8510C analyser was connected to an 8517A S-parameter test set (Keysight, Melbourne, Australia) with an 83651B synthesised frequency source. An IBM compatible computer controls the system, with the software written by Amiet (DSTO, Melbourne, Australia) [12]. The analyzer was calibrated before each measurement. The calibration plane was located at the position of the sample. The calibration was performed without any sample between the two horns, where the transmitted signal corresponds to the total response from the sample and the diffraction. As the free space transmission measurement technique is non-destructive, repeated measurements can be performed on the same sample at set periods of aging. After the initial measurement, readings were taken at 16, 48 and 72 weeks of aging. Changes in the real and imaginary parts of permittivity and reflection, transmission, absorption and total transmission losses during aging are presented and discussed.

## 3. Results and Discussion

### 3.1. The Influence of Aging on Surface Resistivity of PPy-pTSA–Coated Nylon-Lycra

**Figure 4:**
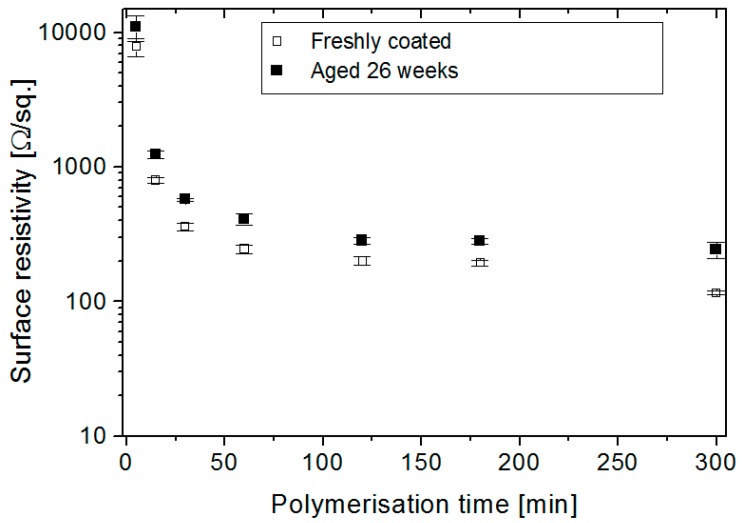
Surface resistivity (R_S_) for newly coated and aged (six months) conducting PPy-*p*TSA–coated Nylon-Lycra as a function of polymerisation time. *p*TSA concentration: 0.018 mol/L.

**Figure 5:**
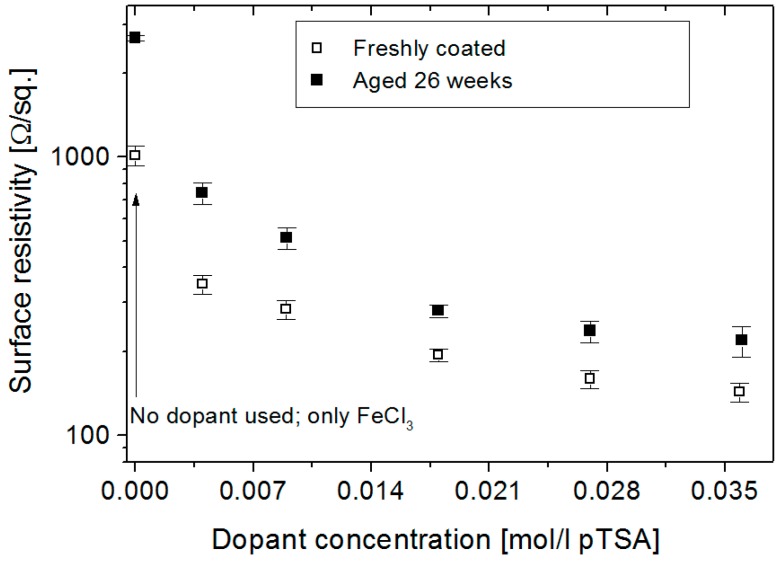
Surface resistivity (R_S_) for recently coated and aged (six months) conducting PPy-*p*TSA Nylon-Lycra as a function of dopant concentration. Polymerisation time: 180 min.

The increase in surface resistivity during the aging of conducting Nylon-Lycra samples of varying polymerisation times and dopant concentrations are shown in Figure 4 and Figure 5.

Short polymerisation times resulted in lightly PPy-coated samples with high surface resistivity. The base fabric was white in colour and the light coating produced fabrics light- to medium-gray in tone and with high surface resistivity. Conversely, longer polymerisation times resulted in thicker coatings deep black in colour with considerably lower surface resistivity. The depth of the tone of the coating is a qualitative indication of its surface resistivity. The surface resistivity decreased with the increase in polymerisation time as seen in Figure 4. There was a rapid decrease in the surface resistivity of around 90% with an extension of the polymerisation time from 5 to 15 min. An increase of the polymerisation time from 15 to 30 min resulted in a further 50% drop, and an additional increase from 120 to 180 min caused an approximately 3% drop in the surface resistivity, finally reaching a plateau at high polymerisation times (Figure 4). Samples aged for six months at room temperature (20 °C, 65% RH) displayed a similar trend of increase in surface resistivity with polymerisation time but with 50%–80% higher resistivity across the polymerisation time range.

Similarly, the surface resistivity decreased with the increase in the concentration of the dopant, reaching a plateau at high concentrations. Even the addition of small concentrations of the dopant *p*-toluene sulfonic acid lowered the resistivity up to 70%, as illustrated in Figure 5. Samples aged for six months displayed a surface resistivity increase of approximately 40% for dopant concentrations of 0.018 mol/L and higher, whilst a much higher increase in surface resistivity was seen in lightly doped samples, with low concentrations of *p*TSA. The increase in surface resistivity upon aging is the largest in the undoped sample (+165%), suggesting that the dopant significantly stabilises the conducting film.

### 3.2. Permittivity Response for Coated Nylon-Lycra during Aging

**Figure 6:**
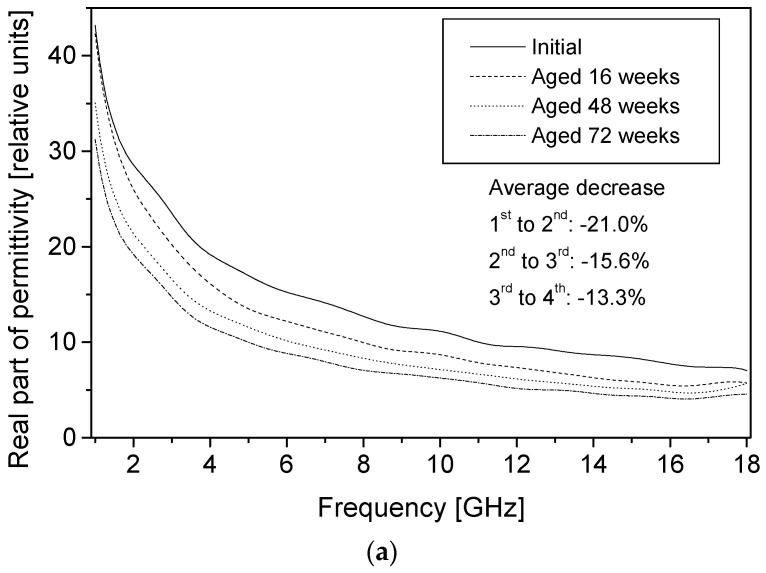

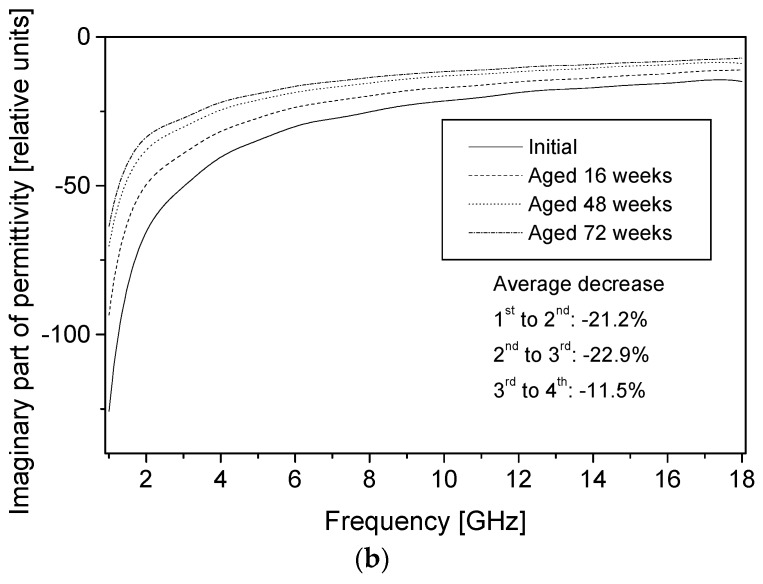
(**a**) Real part of permittivity for PPy-*p*TSA–coated Nylon-Lycra. Polymerisation time: 180 min, concentration: 0.018 mol/L; (**b**) Imaginary part of permittivity for PPy-*p*TSA–coated Nylon-Lycra. Polymerisation time: 180 min, concentration: 0.018 mol/L.

Both the real and imaginary parts of permittivity for newly coated conducting textiles changed upon aging at room temperature (20 °C, 65% RH). Figure 6a,b shows permittivity results for a PPy-*p*TSA–coated Nylon-Lycra with a coating time of 180 min. As the frequency of the radiation increased, magnitudes of both the real and imaginary parts of the permittivity decreased continuously, irrespective of the duration of the aging, dopant concentration and polymerisation times. The response is frequency-dependent, which is often referred to as it being dispersive. The samples showed a smooth permittivity response both when new and aged, indicating that the method used gives reliable values of permittivity. As has been suggested earlier, this also confirms the free space transmission method as being very useful for quality assurance (QA) measurement [13].

**Figure 7:**
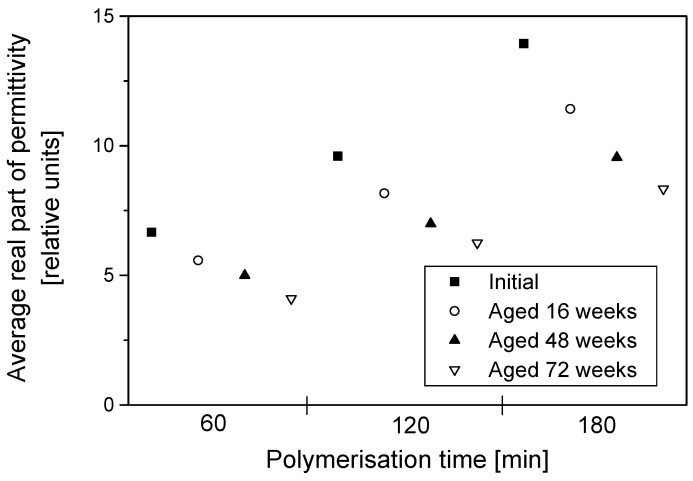
Averages of the real part of permittivity for PPy-*p*TSA–coated Nylon-Lycra during degradation during aging for three different polymerisation times (60, 120 and 180 min). *p*TSA concentration: 0.018 mol/L.

**Figure 8:**
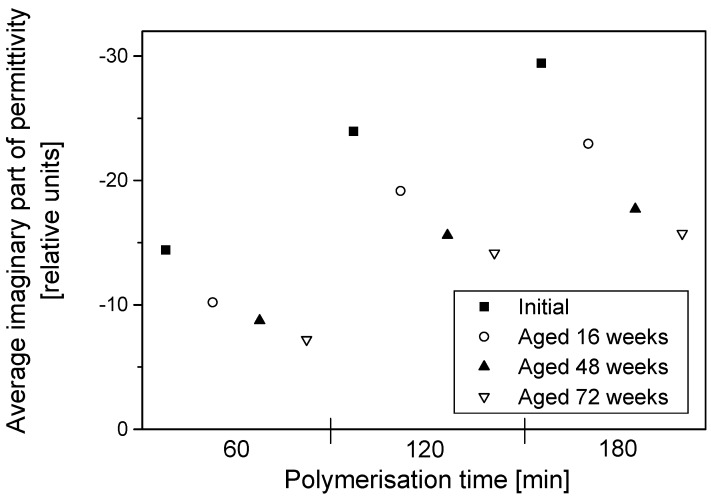
Averages of the imaginary part of permittivity for PPy-*p*TSA–coated Nylon-Lycra during degradation during aging for three polymerisation times (60, 120 and 180 min). *p*TSA concentration: 0.018 mol/L.

Since the imaginary part of permittivity gives an indication of how lossy the material is, it is reasonable to infer that the least conducting of the samples should have the lowest absolute values of the imaginary part of permittivity and vice versa. The electrical resistivity of samples decreased with the increase in dopant concentration and polymerisation time. Nevertheless, during aging, electrical resistivity decreased for all samples irrespective of their dopant concentration and coating times and this manifests as a reduction in magnitude of the imaginary part of the permittivity, as all the samples become less lossy with aging. The change of permittivity with frequency had similar patterns for all the samples, and those with high dopant concentrations and those with thicker coatings displayed higher absolute values of permittivity throughout the frequency range. During aging, the surface resistivity of the textile increased significantly, manifesting as decreased permittivity and lower total transmission loss in all aged samples. A typical example of the degradation of the average real and imaginary part of the permittivity during aging is displayed in Figure 7 and Figure 8, with each section of the graph representing polymerisation times of 60, 120 and 180 min, respectively.

A general look into the change in the permittivity amongst samples from different polymerisation times of 60, 120 and 180 min showed that all samples, irrespective of their coating times, displayed a decrease in permittivity with an increasing aging time. The rate of the decrease of permittivity appeared to level off with the aging time for all polymerisation times. There was FT-IR evidence of the formation of a carbonyl peak that may be partially attributed to the loss of conductivity via perturbation of the PPy conjugation [5]; this in turn would change the nature of the interaction of the material with electromagnetic waves and manifest as a reduction in permittivity as seen in Figure 7 and Figure 8.

### 3.3. Reflection, Transmission, Absorption and Total Transmission Loss for Coated Nylon-Lycra during Aging

**Figure 9:**
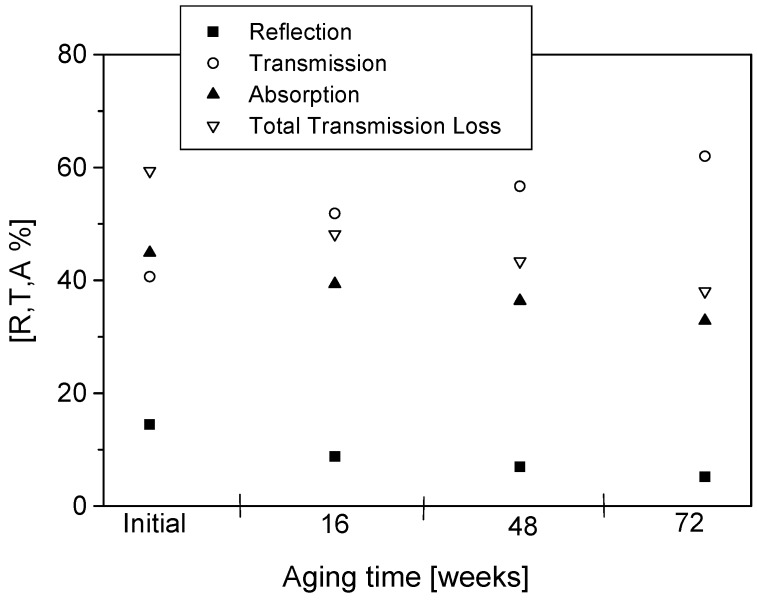
Reflection, transmission, absorption and total transmission loss for PPy-*p*TSA–coated Nylon-Lycra. Polymerisation time: 60 min, *p*TSA concentration: 0.015 mol/L. Please make the explanations smaller and put them inside the piture like the Figure 5, Figure 6, Figure 7, Figure 8 and Figure 9.

**Figure 10:**
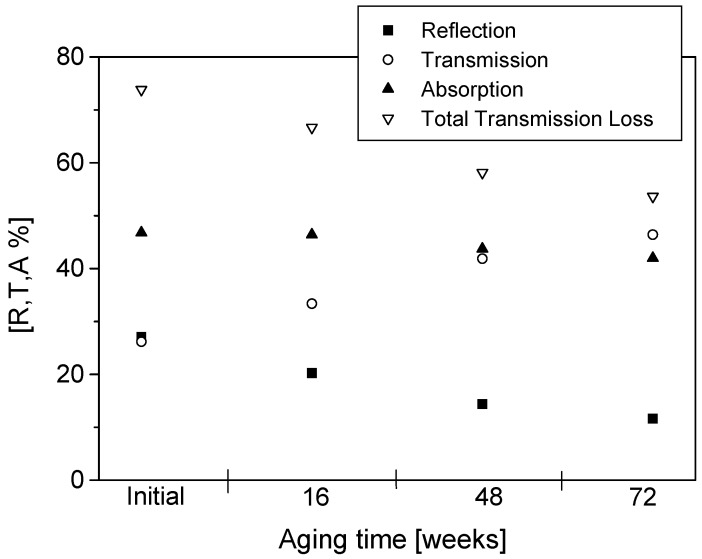
Reflection, transmission, absorption and total transmission loss for PPy-*p*TSA–coated Nylon-Lycra. Polymerisation time: 180 min, *p*TSA concentration: 0.015 mol/L.

**Figure 11:**
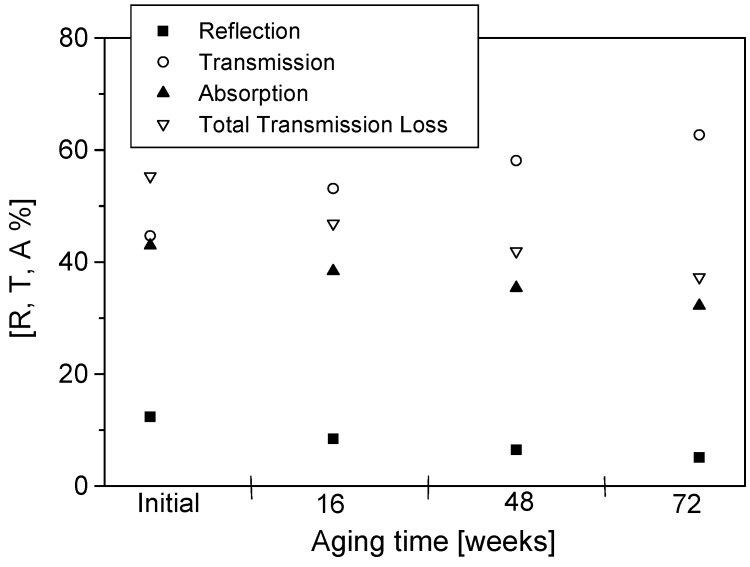
Reflection, transmission, absorption and total transmission loss for PPy-*p*TSA–coated Nylon-Lycra. Polymerisation time: 60 min, *p*TSA concentration: 0.027 mol/L.

**Figure 12:**
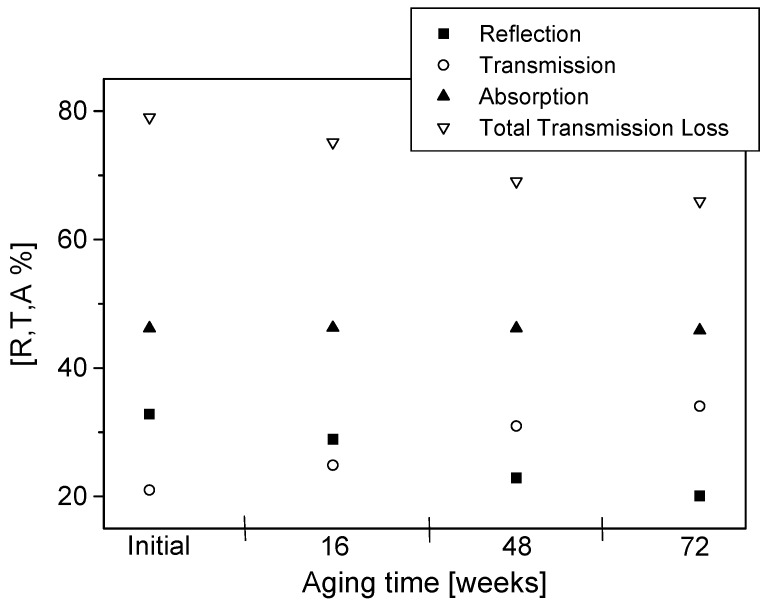
Reflection, transmission, absorption and total transmission loss for PPy-*p*TSA–coated Nylon-Lycra. Polymerisation time: 180 min, *p*TSA concentration: 0.027 mol/L.

The percentage reflection (R), transmission (T) and absorption (A) as well as total transmission loss initially and after 16, 48 and 72 weeks of aging are presented in Figure 9, Figure 10, Figure 11 and Figure 12. The effects of conducting polymer coating times, dopant concentration and aging can be seen in these figures. In a study of the aging of free-standing PPy films, it was shown that shielding effectiveness reduced up to 60% for lightly doped films after 24 months of aging [14,15,16]. Similar results have been reported for polyaniline [17], displaying a very rapid degradation at early stages of aging, only to slow down at longer aging times.

The PPy-coated Nylon-Lycra with a *p*TSA concentration of 0.015 M exhibited total transmission losses of 37% (Figure 9) and 26% (Figure 10) for samples coated for 60 min and 180 min, respectively, upon aging for 72 weeks at room temperature (20 °C, 65% RH). The lower transmission loss upon aging of samples coated with PPy for longer coating times is attributed to the thicker polymer depositions due to increased reaction times. Although the conductivity of the fabric reached a plateau with the increasing polymerisation time, the prolonged coating time causes thicker PPy coatings and a thorough penetration of the polymer into the structure of the fabric, thus reducing the microwave transmission whilst increasing the reflection. Not only the polymerisation time but also the concentration of the dopant has an important effect on the insertion loss. The sample with a higher dopant concentration of 0.027 mol/L *p*TSA had a transmission loss of 32.6% for short polymerisation times (Figure 11) and 16.5% for long polymerisation times upon aging for 72 weeks (Figure 12). This is in accordance with previous investigations [4] that indicated that highly doped specimens had lower losses in electrical resistivity, hence displaying better stability of shielding effectiveness. Conversely, the lightly *p*TSA-doped specimens were more unstable. A high dopant concentration and/or longer polymerisation times are more effective in reducing microwave transmission and improving the longevity of the electrical properties. It is also interesting to note that the aging occurred at a slower rate in the conducting textiles than observed in the free-standing films.

It can be observed from Figure 9, Figure 10, Figure 11 and Figure 12 that reflection values decreased at a higher rate than the absorption values. For polymerisation times of 60 min, the average decrease in the reflection between subsequent measurements (16 weeks) was 25% while the average decrease in absorption was around 9%. For the samples with a longer polymerisation time of 180 min the trend was the same: the average reflection decrease was 19.7% whereas the average absorption decrease was only 1.64%. These results reinforced the observations that the samples with longer polymerisation times and higher dopant concentrations had better stability than samples with short polymerisation times. It was also observed that the sample made with only FeCl_3_ acting as the oxidant/dopant had much lower stability against the degradation of microwave properties than all the samples with added *p*TSA as the dopant. The percentage reflection in the sample made with only FeCl_3_ decreased twice as rapidly compared with all samples with added dopant. The absorption levels dropped almost three times as fast. An increase in the *p*TSA concentration slowed down the rate of decrease of both the reflection and absorption. A sufficiently high concentration of dopant (greater than 0.027 mol/L) even caused an increase in the absorption values during the first eight weeks of aging. The same was true for very long polymerisation times of 240 min or 300 min. The preserving effect of the dopant *p*TSA was noted in all the measurements.

## 4. Discussion and Conclusions

The permittivity of PPy-coated textiles was measured using a free space transmission measurement technique over the frequency range of 1–18 GHz. Results showed that the conducting polymer-coated textiles were lossy over the full frequency range. As the frequency of radiation increased, magnitudes of both the real and imaginary parts of the permittivity decreased continuously irrespective of the duration of the aging, dopant concentration and polymerisation times. The measurements were relatively free from diffraction aberrations, especially for large sample sizes. The permittivity increased with the polymerisation time but stabilised after 120 min. The change in the real part of the permittivity was not significant beyond 12 GHz, regardless of the polymerisation time, and during aging, electrical resistivity decreased for all samples, irrespective of their dopant concentration and coating times, and the increased resistivity manifested as a reduction in the magnitude of the imaginary part of the permittivity due to samples becoming less lossy with aging. The dopant not only improved the electrical and microwave properties of the fabrics but also proved to have a stabilising effect against aging. Both the real and imaginary parts of the permittivity remained stable above dopant concentrations of 0.018 mol/L *p*TSA. The levels of absorption were higher than the levels of reflection in all the tested samples. This is attributed to the relatively high resistivity of the PPy-coated fabrics. The absorption level is relatively even throughout the frequency range for different concentrations of dopants and polymerisation times. In metallic materials, the electromagnetic interference shielding is very high and exclusively attributed to the reflection of radiation due to the high conductivity of the material. In medium-level conductivity materials, a large part of the radiation is absorbed in the material and dissipated as heat. The conducting textiles investigated here both reflected and absorbed the incident microwave radiation.

The shielding effectiveness of conducting textiles increased slightly as the frequency increased. This is a result of the increased contribution to shielding from reflection, which is more frequency-dependent than the absorption. The absorption showed a maximum in the 4–7 GHz range. Both the dopant concentration and polymerisation time affected the total shielding effectiveness and it was difficult to distinguish either of these two factors as being exclusively deterministic of shielding behaviour.

Topographic features of the actual coating were not considered to influence the shielding effectiveness of these fabrics as high frequencies allowed little or no interfacial polarisation to take place in the material. The shielding analysis showed that chemical structure influences the shielding effectiveness since a higher dopant concentration resulted in a higher conductivity and, hence, a higher transmission loss. The absorption-dominated, considerably high shielding proves that these conductive textiles are good light-weight microwave absorbers.
